# Ectopic meningioma in the bilateral nasal olfactory cleft: A case report and literature review

**DOI:** 10.3892/ol.2015.2970

**Published:** 2015-02-17

**Authors:** YI ZHANG, WEI-QIANG TENG, XIAO-PING CHEN, JIAN WU

**Affiliations:** 1 Department of Otolaryngology, Shanghai Pudong New Area Gongli Hospital, Shanghai 200135, P.R. China; 2 Department of Otolaryngology, Postgraduate Education College, Ningxia Medical University, Yinchuan, Ningxia 750004, P.R. China; 3 Department of Pathology, Shanghai Pudong New Area Gongli Hospital, Shanghai 200135, P.R. China

**Keywords:** meningioma, ectopic therapy, nasal polyp diagnosis

## Abstract

Certain nasal neoplasms, such as ectopic menigioma, present as nasal polyps, together with similar symptoms. The present study reports the diagnosis and treatment of ectopic meningioma in the bilateral nasal olfactory cleft in order to improve the diagnosis and treatment of ectopic meningioma in the nasal cavity. By retrospectively analyzing the clinical data and reviewing the associated literature, a detailed introduction to the clinical manifestation, diagnosis and treatment of ectopic meningioma of the nasal cavity was ascertained. The tumor was removed from the sinus by functional endoscopy surgery. Regular follow-up appointments were scheduled every three months, with no evidence of recurrence to date. The olfactory recovery and nasal ventilation were normal subsequent to surgery. Meningiomas are infrequently occurring tumors with unpredictable clinical behavior. A clear understanding of the etiology and appropriate diagnostic and management principles may aid in overcoming the challenges of treating primary extracranial meningiomas.

## Introduction

Primary and secondary ectopic meningiomas are rare lesions (1). In addition, primary ectopic meningiomas that develop in the nasal sinus or nasal cavity possess an unknown etiology (2).

Meningiomas account for 10–15% of all intracranial tumors (3), but primary and secondary meningiomas outside the central nervous system are uncommon. The most common sites of extracranial meningiomas are the skull, scalp, nose, orbit, paranasal sinuses, middle ear, neck and skin (4).

Extracranial meningiomas of the sinonasal tract are rare tumors that are frequently misdiagnosed, resulting in inappropriate clinical management. Primary extracranial meningiomas are likely to arise from the transformation of embryonic arachnoid cell remnants of ectopic meningocytes, which are derived from pluripotent mesenchymal cells. The diagnosis of extracranial primary meningiomas requires confirmation by computed tomography (CT) to exclude the presence of an intracranial mass or any underlying bony erosion of the skull base (5). Treatment usually involves tumor resectioning, and additional treatment with radiotherapy and chemotherapy. Fine-needle aspiration cytology of the lesion can be inaccurate, and a final diagnosis is usually made on the basis of a histological examination of the excised mass (6). The present study reports the diagnosis and treatment of a nasal meningioma and reviews the recent literature. Written informed consent was obtained from the patient.

## Case report

A 60-year-old female presented to the Otolaryngology Department of the Shanghai Pudong New Area Gongli Hospital (Shanghai, China) with hyposmia that had been present for a six-month period, without rhinorrhea or epistaxis. Clinical examination did not identify a nasal obstruction, and the patient did not possess a history of headaches or visual problems. Endoscopy identified the presence of a neoplasm in the bilateral olfactory cleft, with the nasal polyp morphology necessitating a radiological examination.

**Figure 1:**
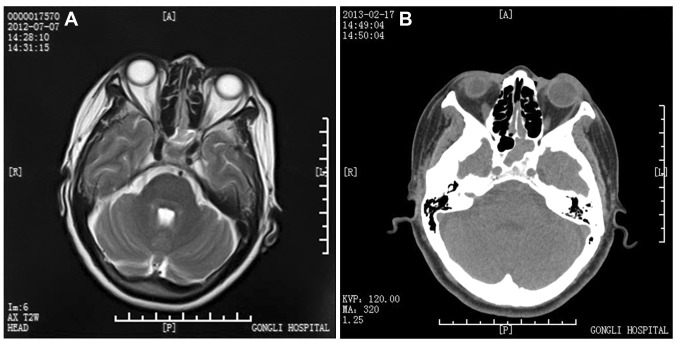
Axial magnetic resonance imaging/computed tomography (MRI/CT) scan. (A) Axial T2 weighted MRI scan of the head revealing a soft tissue mass situated exclusively in the bilateral olfactory cleft area, not invading other sinuses, orbit, or the bone structure of the skull base. (B) A 64-slice spiral axial CT plain scan of the paranasal sinus in the same area described in A.

A magnetic resonance imaging (MRI) examination in the Radiology Department of Shanghai Pudong New Area Gongli Hospital revealed sphenoid sinusitis on the left side of the olfactory cleft and a high-density shadow in the bilateral olfactory cleft five months prior to presenting at the Otolaryngology Department, where a CT scan of the paranasal sinus was conducted. Radiological examination of the nasal cavity prior to surgery identified the presence of soft tissue shadows in the bilateral olfactory cleft area and sinusitis in the left side of the sphenoid. MRI and CT scans of the orbit and nasal cavity revealed that the soft tissue mass was situated exclusively in the bilateral olfactory cleft region and did not invade other sinuses or the orbit (Fig. 1A and B).

**Figure 2:**
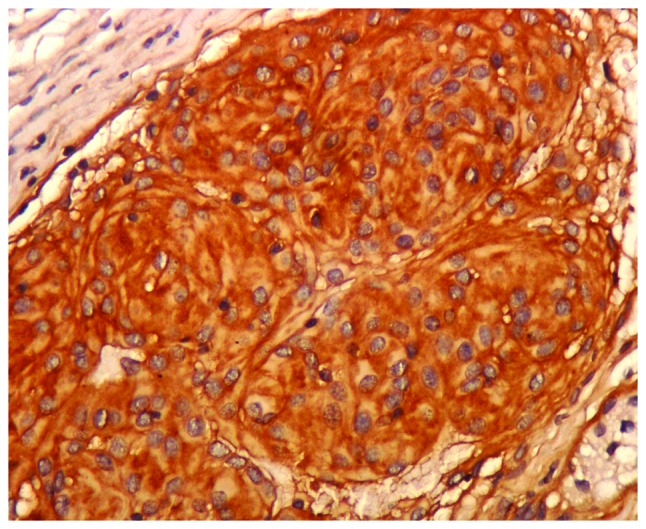
Immunostaining of the excised meningioma tissue for EMA. The tumor cells varied in size, exhibited a high nucelo-cytoplasmic ratio, and demonstrated positivity for EMA. Spiral structures were observed, however, no psammoma bodies were identified. The cells were stained with hematoxylin and eosin and viewed under a magnification of ×40. EMA, epithelial membrane antigen.

**Figure 3:**
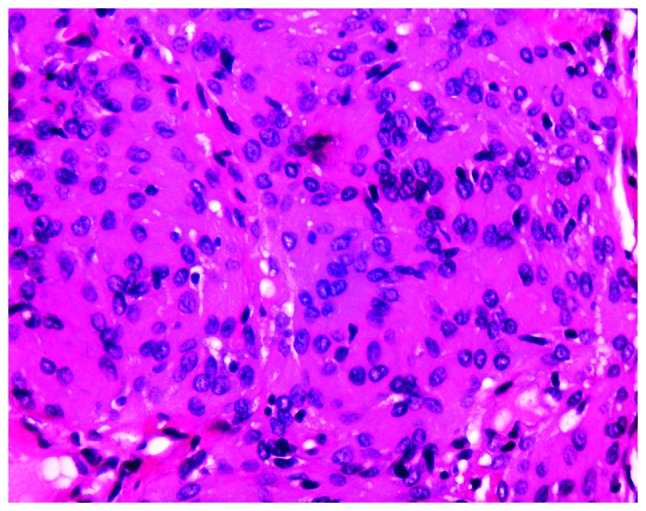
Microsopic analysis. The cells demonstrated a lobulated arrangement and were spaced along collagen fibers. The tumor cells also varied in size. The cells were stained with hematoxylin and eosin and viewed under a magnification of ×40.

The histopathological appearance of the lesion was meningothelial, a common tumor type in which the cells present in a lobulated arrangement and are spaced along collagen fibers. The tumor cells also varied in size. They demonstrated a high nucleocytoplasmic ratio and a marginal oval nucleus, with nucleoli discernable in each cell. Fibroblasts were arranged in a matrix rich in collagen and crosslinking reticular fibers. Spiral structures were visible and did not contain psammoma bodies. Mitosis was inconspicuous, and no necrosis was observed (Figs. 2 and 3).

Immunohistochemical examination identified the presence of the epithelial membrane antigen expression. However, the immunoreactivity was focal and generally weak, identifying only a small number of positive cells in the sample examined. Other epithelial markers were also identified, including the S-100 protein in the nucleus and the cytoplasm. Isolated tumor cells were stained for the expression of Ki-67 in the nucleus.

The tumor was excised from the sinus by functional endoscopy surgery. The patient continues to experience disease-free survival seven months subsequent to surgery. Post-operative nasal endoscopic examination revealed no residual mass. Follow-up visits were scheduled every three months, with no evidence of recurrence to date. The patient’s olfactory recovery and nasal ventilation were normal subsequent to surgery.

## Discussion

The present patient presented with a benign primary ectopic meningioma. Meningiomas of the nasal cavity, particularly in the olfactory cleft, are rare. Primary ectopic meningiomas are challenging to diagnose, partly due to the infrequency of their presentation and often being confused with nasal polyps. However, primary ectopic meningiomas tend to be aggressive. Definitive diagnosis requires histological examination. Anosmia is among the first symptoms associated with ectopic meningioma, although patients also present with headaches or visual problems (7). Endoscopic surgery to remove the growth is an accepted therapy for this condition and is associated with a good survival rate (2).

Primary extracranial meningiomas of the head and neck are rare, with the majority presenting at secondary locations relative to the primary intracranial tumor. Primary extracranial meningiomas account for 1–2% of all meningiomas and are generally associated with a favorable prognosis. Subsequent to the diagnosis of a meningioma, it is necessary to exclude meningioma of the neuraxis or a primary central meningioma (9).

The most frequent extracranial sites reported for primary meningioma are the nasal cavity, paranasal sinuses, cranial bones, middle ear, scalp and soft tissues of the face and neck, and the parotid gland (10). The largest study that reported extracranial head and neck meningiomas consisted of 146 cases (8), among which the majority of meningiomas were of the skin and scalp (n=59), middle ear (n=26), nasal cavity (n=17), temporal bone (n=2), or the parotid gland (n=1). Other similar studies included patients with meningioma of the sinonasal tract (n=30) (11), ear and temporal bone.

The etiopathogenesis of extracranial meningiomas involves the migration of arachnoid cells derived from the neural crest. However, additional mechanisms have been proposed. For example, extracranial meningiomas may potentially originate from: i) Arachnoid cells of nerve sheaths emerging from the skull foramina; ii) pacchionian bodies that have been displaced or entrapped in an extracranial location during embryological development; iii) arachnoid islets that have been displaced due to trauma or cerebral hypertension; and iv) undifferentiated mesenchymal cells (12).

The classification system utilized by Hoye *et al* (13) encompasses the major etiologies suggested in the development of extracranial meningiomas: i) Extracranial extensions of a meningioma with an intracranial origin (secondary); ii) extracranial extensions of a meningioma arising in a neural foramina (primary); iii) ectopic, without any connection to intracranial structures (primary); and iv) extracranial metastasis from an intracranial meningioma (secondary).

The meningioma described in the present study was derived from the third group of the aforementioned classification system. The presentation of hyposmia in the current patient facilitated the diagnosis of meningioma prior to the invasion of the tumor into other sinuses or the orbit.

The current World Health Organization classification distinguishes between three grades of meningioma: Typical or benign (Grade I); atypical with frequent mitosis (Grade II); and anaplastic with invasion (Grade III). However, Zulch (14) suggested that the completeness of extirpation is more important than histological grading for classifying meningioma. Although meningiomas are classified as benign, since they do not metastasize, they demonstrate a predilection for invading crevices and foramina, and necrosis may be spread between one cavity and another (15). In the present study, the benign nature of the tumor was evident from its slow growth and non-involvement with the orbit or any cranial nerves. Radiological examination provided precise information regarding the extent of tumor invasion, which is key to diagnosis. However, confirmation of the radiological diagnosis by histological and immunohistochemical examinations was required.

Rushing *et al* (8) reported in 2009 that 76% of extracranial meningiomas were progesterone receptor-positive, 96% were somatostatin receptor-positive, 89% were epidermal growth factor receptor-positive, and 19% were estrogen receptor-positive (Table I) (8). Accordingly, Tamoxifen and RU-486, an antiprogesterone, are being investigated for potential as treatments for certain meningiomas. Monosomy 22 is a characteristic and frequent chromosomal aberration associated with meningioma and its prognosis (16).

Radical surgical resection has been associated with a good prognosis in patients with meningioma, but the benefit of adjunctive post-operative radiotherapy has not yet been established (3). Radiotherapy is suggested as the treatment of choice for unresectable malignant meningiomas and for recurrent meningiomas in patients with a late onset or other conditions that do not permit surgery to be conducted, including poor general condition and coagulation disorders.

Novel techniques available for meningioma management include proton irradiation and stereotactic radiosurgery using a gamma knife. Incomplete tumor removal, atypical or malignant tumors, nucleolar prominence, more than two visible mitotic events per 10 high-power fields and a heterogeneous contrast enhancement on CT are associated with increased rates of recurrence (17).

Meningiomas are infrequently occurring tumors with unpredictable clinical behavior. A clear understanding of the etiology and the appropriate principles for the diagnosis and management of these lesions may aid in overcoming the difficulty of treating primary extracranial meningiomas. The present case revealed that CT and immunohistochemical examination are important for the diagnosis of ectopic meningioma of the nasal cavity and that tumor resection presents the optimum treatment for meningiomas.

## Figures and Tables

**Table I:** Immunohistochemical profile of extracranial meningiomas.

Antigen	Total number of observations, n	Number of positive reactions, n (%)
Vimentin	78	78 (100.0)
Epithelial membrane antigen^a^	80	61 (76.3)
Cytokeratin^b^	75	18 (24.0)
CK7	55	12 (21.8)
S-100 protein	78	15 (19.2)
CAM 5.2	54	3 (5.6)
Synaptophysin	75	3 (4.0)
CK20	52	1 (1.9)
GFAP	69	1 (1.4)
Chromogranin	72	0 (0.0)
Synuclein	18	0 (0.0)
Ki-67 index >1%	78	21 (26.9)

a (8). Stained with epithelial membrane antigen stain;

b Stained with AE1/AE3 and CK1.

CK, cytokeratin; GFAP, glial fibrillary acidic protein.
